# Role of the advanced nurse practitioner within the vascular team: A qualitative study of vascular physicians and nurses

**DOI:** 10.3389/fpubh.2023.1070403

**Published:** 2023-03-30

**Authors:** Thibaut Kubiak, Jonas Sitruk, Andréanne Durivage, Lina Khider, Nassim Mohamedi, Grégoire Détriché, Emmanuel Messas, Tristan Mirault, Guillaume Goudot

**Affiliations:** ^1^Vascular Medicine Department, Georges-Pompidou European Hospital, Assistance Publique–Hôpitaux de Paris (AP–HP), Paris, France; ^2^Université Paris Cité, INSERM U970 PARCC, Paris, France; ^3^Faculty of Medicine and Health Sciences, University of Sherbrooke, Sherbrooke, QC, Canada

**Keywords:** advanced practice, advanced nurse practitioner, cardiovascular prevention, vascular medicine, peripheral artery disease, healthcare circuit, nursing

## Abstract

**Objective:**

To assess the perception of Advanced Nurse Practitioners (ANP) by physicians and nurses in vascular medicine. As the status of ANP in France was recently enacted by law in 2018, we aimed to investigate physicians and nurses working with patients suffering from Peripheral Artery Disease (PAD) to gather their opinions and draw the cooperation outlines these practitioners could have with an ANP.

**Methods:**

A qualitative study based on in-depth interviews was conducted among healthcare practitioners taking care of patients with PAD: 10 physicians working either in a private practice settings or hospital settings or both, and eight nurses working within a hospital inpatients vascular unit. Verbatim responses were extracted and coded according to a continuous thematization method.

**Results:**

Three main features emerged from participants’ responses. Vascular medicine has a specific organization with a significant lack of time and staff to fulfill the mission regarding patients’ severity of illness. Second, the ANP is wanted to fill part of this gap. The expected benefits include a smoother care pathway and increased capacity for cardiovascular education and prevention, especially during consultations. Lastly, some clarification is required to integrate such new practitioners within vascular teams already in place.

**Conclusion:**

Advanced nurse practitioners could be the missing link in a “Vascular team” by creating a continuum in the care of patients with PAD, ensuring clinical assessment, nursing supervision, adverse event screening, and renewing drug prescriptions with the required adaptations while ensuring essential part of therapeutic education adapted to each patient.

## 1. Introduction

### 1.1. Background

Advanced Nurse Practitioner (ANP) treats and diagnoses illnesses, advises the public on health issues, manages chronic disease, and engages in continuous education (1). While ANPs have been present for years in English-speaking countries, the status of ANPs in France was only enacted by law in 2018 (2).

The International Council of Nurses released guidelines in April 2020 for advanced nursing practice (1). According to these guidelines, advanced practice nursing extends the boundaries of nursing practice, contributes to nursing knowledge, and requires additional education at a minimum of a master’s level. Advanced practice nurses provide safe and competent patient care, and have measurable levels of practice and competency. In France, the act states that the ANP participates in the overall management of a patient whose follow-up is entrusted to him or her by a physician and for whom the therapeutic choices have been clearly defined by the physician. There are currently five areas of specialization, including stable chronic diseases, prevention, and multiple common diseases in primary care. The ANP has completed an additional 2 years of training (master’s degree), which has provided him with specific skills and knowledge in the medical field. These skills enable him/her to perform advanced tasks such as clinical assessments, technical procedures, and prescriptions. The ANP regularly follows patients referred by a physician for follow-up of their pathologies. He/she collaborates with the team to discuss patient cases and may call on the physician when the limits of his/her scope of practice are reached.

In the cardiovascular field, peripheral artery disease (PAD), one manifestation of atherosclerotic cardiovascular disease, affecting approximately 238 million people worldwide, is an archetypal condition, combining several chronic pathologies (3). 80% of patients with PAD have hypertension, two-thirds hyperlipidemia, almost one-half diabetes mellitus, and at least one-third still smoke (4–6). Furthermore, these patients are at very high cardiovascular risk, putting them at greater risk of a cardiovascular event than the risk of progression of lower extremity arterial disease (7). Vascular medicine faces the challenge of improving their care and quality of life, but some goals cannot be achieved for every patient. Indeed, patients do not always benefit from the treatments recommended by the learned societies. The reasons raised by the various studies are non-compliance with medication, therapeutic inertia, and non-prescription of recommended treatments. Patients with PAD could benefit from ANP care, but there is little published on the role of ANPs in vascular medicine worldwide to date ANP (8, 9), and none in France.

## 2. Methods

### 2.1. Aim

We wished to explore the problems encountered by physicians and nurses in vascular medicine, particularly with patients with PAD, and to gather their views on whether an ANP could be involved in addressing them. With the goal of answering the following question: How could an ANP be complementary to vascular medicine physicians for the management of patients with PAD in particular?

### 2.2. Design

We conducted an in-depth multi-center exploratory qualitative study (in the city of Paris and close neighborhoods, with one respondent in the Provence region) using semi-structured interviews from February through April 2021, with thematic analysis.

### 2.3. Participants

Two categories of practitioners taking care of patients with PAD have been selected: vascular medicine physicians working either in private practice, hospital, or both settings, and nurses working within a hospital in-patient vascular medicine unit. We solicited doctors and nurses from different vascular medicine departments in Paris and its surroundings by e-mail and then asked participants about the recommendations of the professionals interviewed. To respect the principle of maximum variation to create the sample, investigators varied the characteristics of the participants: number of years of practice, age, gender, working experience with ANPs, knowledge about the ANP profession, place, and type of practice.

### 2.4. Data collection

We used semi-structured interviews to collect data from professionals directly involved in caring for patients with PAD. This method allows us to explore “the thoughts, feelings, and beliefs of the participants on a particular subject” (10). We used a semi-structured interview tool based on previously published guidelines (11). TK conducted all interviews in French, using the semi-structured interview guide that included an introduction introducing the study and its purpose, 10 questions, and then a section describing ANPs so that all interviewees would have the same basic information about this new profession, regardless of their experience with ANPs (Supplementary Data 1). Interviews were conducted face-to-face at their place of employment and by telephone when face-to-face interviews were not possible. All interviews were audio recorded.

### 2.5. Ethical considerations

The investigators subjected each participant to a verbally informed consent process. There was no known relationship between the investigator and the participants. Participants were informed of their right to withdraw from the study at anytime. All interviews were audio-recorded and then transcribed and anonymized. After transcription, the recordings were erased.

### 2.6. Data analysis

The thematic analysis was carried out using NVIVO 12^®^ software (QST International^®^, Australia), which allows verbatim responses to be extracted and coded according to a continuous thematization method. We followed a six-phase method (12):

1. Familiarizing yourself with your data
2. Generating initial codes
3. Searching for themes
4. Reviewing themes
5. Defining and naming themes
6. Producing the report

### 2.7. Validity and reliability

The quality control of the study was carried out using the Consolidated Criteria for Reporting Qualitative Studies (COREQ) list (13), with all items completed (Supplementary Data 2). Three investigators (TK, JS, and GG) read each transcript and worked together to create a coding structure and codebook. The coding team met regularly to discuss the meaning and application of the codes. New themes that had emerged and those that needed revision were discussed, and the codebook was updated accordingly.

## 3. Results

**Figure 1:**
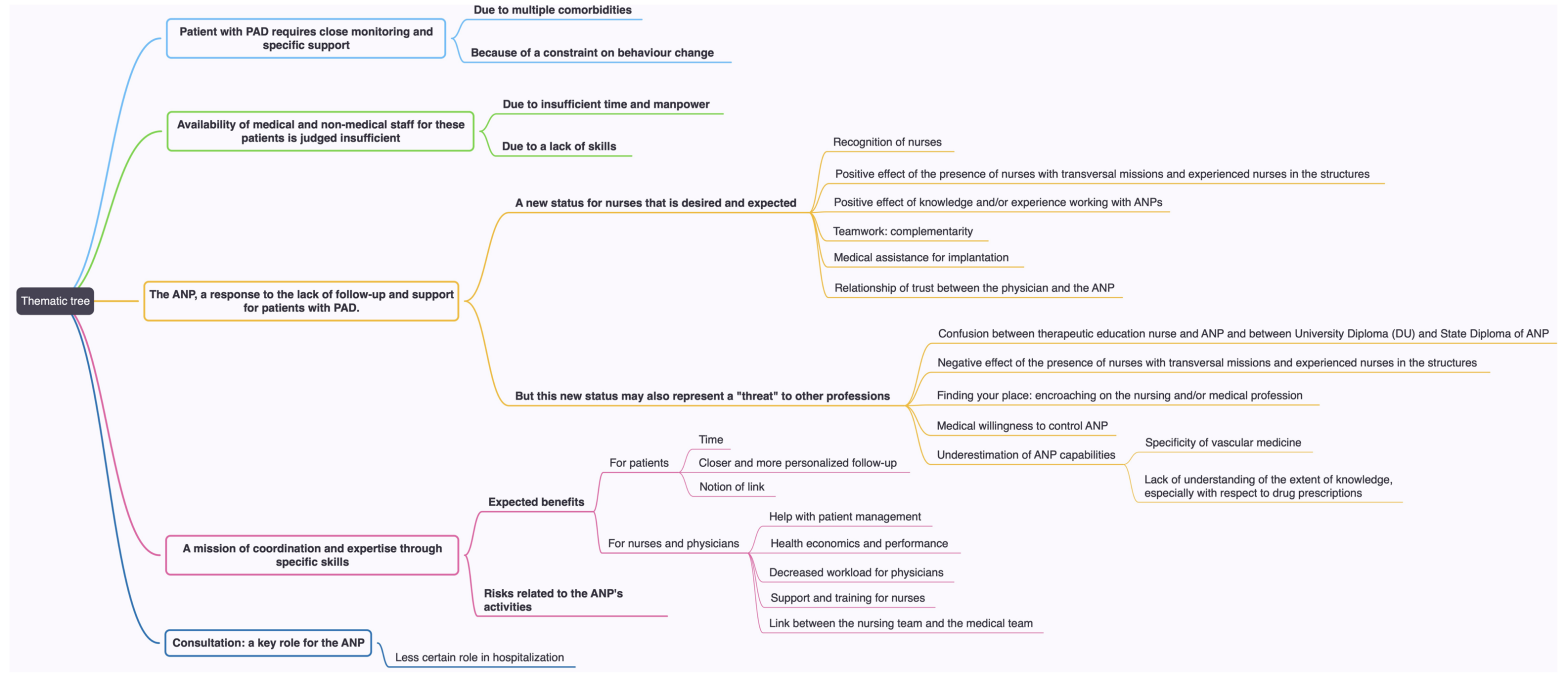
Thematic tree based on the data analysis. ANP, advanced nurse practitioner; PAD, peripheral artery disease.

Eighteen interviews were conducted to reach data saturation and create a sample of 18 healthcare professionals divided into 10 vascular physicians and eight nurses. Data saturation is reached when the interviews and their analysis do not yield any new ideas or themes (14). Data saturation was reached at the end of the eighth interview with confirmation by two additional interviews for physicians and at the end of the seventh interview with confirmation by one additional interview for nurses. Table 1 illustrates the number and type of respondents included in this study. The data analysis allowed the creation of a thematic tree (Figure 1).

**Table 1:** Vascular medicine physicians and nurses participating in the study.

Physician (P) Nurse (N)	Age (year)	Gender	Practice setting	Region	Interview duration (min)
P1	32	W	Public hospital	Ile de France	38
P2	29	W	Public hospital	Ile de France	30
P3	35	W	Private practice	Ile de France	23
P4	34	M	Public hospital and private practice	Ile de France	20
P5	55	W	Public hospital	Ile de France	46
P6	42	M	Public hospital	Ile de France	41
P7	31	W	Public hospital	Ile de France	33
P8	33	M	Public hospital	Ile de France	25
P9	33	W	Private practice	Provence—Alpes—Côte d’Azur	36
P10	31	M	Public hospital	Ile de France	22
N1	51	W	Public hospital	Ile de France	45
N2	25	W	Public hospital	Ile de France	18
N3	53	W	Public hospital	Ile de France	23
N4	34	M	Public hospital	Ile de France	28
N5	27	W	Public hospital	Ile de France	16
N6	30	W	Public hospital	Ile de France	21
N7	27	W	Public hospital	Ile de France	31
N8	33	W	Public hospital	Ile de France	23
In summary					
10 physicians and eight nurses	Median age 33 [25–55]	72% women	15 public hospital, two private practice, and one both settings	Two regions, seven different centers	Median duration 26.5 [16–46]

W, woman; M, man, results are expressed as median [25–75th percentile].

### 3.1. Patient with PAD requires close monitoring and specific support

#### 3.1.1. Due to multiple comorbidities

Patients are often described by their multimorbidity and frailty, with a specific bio-psycho-social profile: Quote of physician number 6 (P6) *“Patients are from less privileged socio-professional classes…with less willpower in general; smoking cessation is more difficult; physical activity is more difficult; they are more sensitive to weight gain and have difficulty reversing the situation.”*

*N4 “*…*they have multiple pathologies so they often have other risk factors, and also other pathologies that are associated, so they can quickly become complex patients*…*”*

It is difficult for the patients to fully understand the relationship between silent cardiovascular risk factors such as hypertension, hyperlipidemia or diabetes mellitus and their symptoms due to atherosclerotic arterial lesions. Especially as the latter is also developing insidiously, leading to a trivialization of the risk. Interventional procedure is often perceived as the key solution: *P10 “According to them, they claim that stenting is the end of the problems and I [the patient] can walk again*.”

#### 3.1.2. Because of a constraint on behavior change

According to the responders, patients do not necessarily feel sick apart from the episodes of pain or trophic disorders. They are described as being reluctant to make even minimal changes to their lifestyle, and some experience isolation due to their illness. This may explain their lack of compliance, particularly when returning home after hospitalization. *N1*: *“They always come back with the same story. They have not stopped smoking; the arteries keep getting obstructed.”*

### 3.2. Availability of medical and non-medical staff for these patients is judged insufficient

#### 3.2.1. Due to insufficient time and manpower

Vascular physicians noted that the delay before the outpatient consultations is too long and the time allowed per consultation too short, making the goals of secondary cardiovascular prevention impossible to achieve. *P4 “Consultations are short, we do not necessarily have time to do all the things we would like to do. (silence). Even if it’s only for therapeutic compliance, for the optimisation of his care.”*

One reason given for the lack of medical time allocated is the fact that vascular medicine is a recent specialty in France with few specialists available to take care of patients with PAD. Previously general practitioners and vascular surgeons were the medical pair in charge of these patients, and the medical time allocated was even less. Yet, one of the responders stated that the training of future vascular medicine specialists would make it possible to form a network to monitor these patients. Currently, university hospitals must ensure the entire follow-up of chronic patients due to the lack of relays in private practice. In the medical wards too, nurses complain of the lack of time for patients, especially in prevention and education: *N3 “*…*we do not have enough time to go further, to consider a follow-up of patients*.*”*

General practitioners are overwhelmed with many patient’s follow-up and the consultations are too short to ensure optimal care for patients with PAD by addressing all the patient-specific elements in a customized manner during a single consultation: *P7* “*A general practitioner for any pathology has 15 min and he cannot go into the details of each patient, …, In other words, medication, blood tests, results, there is perhaps less time for therapeutic education and not for adapting to each patient*.”

#### 3.2.2. Due to a lack of skills

Post-operative vascular surgery follow-up of patients with PAD is emphasized: *P8 “*… *many patients who are hospitalized in the vascular surgery department and who leave the hospital do not necessarily have a statin prescription, and even rarer an Angiotensin-Conversion Enzyme Inhibitors prescription.”* The nurses suggested that cardiovascular prevention is not sufficiently emphasized by physicians who are more focused on their therapeutic activities. Medical time allocated to educational purposes is sparse; one reason is that there is not enough financial return to match the necessary investment. Responders also acknowledge that motivational interviewing is not fully mastered by physicians and nurses to help patients adopt healthy lifestyle: *P3 “*…*and above all I do not know how to do it. I go round in circles. That is to say that I have difficulty* … *I am not good at education*.” *P1 “*…*we do not know the hygiene and dietary measures as well as you (ANP), so we just throw out what we know.“*

Insufficient coordination between different specialists is a problem widely discussed by the responders. Patients are often followed by several specialists with multiple prescriptions, with a risk of iatrogenic medication. The rate at which practice guidelines evolve, the lack of time and updated scientific information can lead to drug renewals without adaptation to the expected therapeutic targets: *P7 “*…*too many physicians are also not necessarily comfortable with the targets set by the guidelines*.” Out-of-hospital follow-up is insufficient according to the nurses, and unscheduled hospitalisations have a significant medico-economic impact. *N4 “the follow-up of patients returning home is not adequately monitored and adapted.”*

### 3.3. The ANP, a response to the lack of follow-up and support for patients with PAD

#### 3.3.1. A new status for nurses that is desired and expected

The creation of this new status in Nursing in France is considered late by many physicians. *P5 “*…*we had to make progress in terms of the responsibility we gave to the nursing teams.”*

According to the nurses, this new status would make the nursing profession more attractive by broadening the field of competencies. Advanced practice is seen as a step forward for nurses, a recognition of their expertise and a broadening of their skills: *N7 “A bit more autonomous compared to us (registered nurses) who are on the ward.”* Most of the responders have a partial knowledge of the ANPs, and, for some of them, a broader vision of the missions: *N3 “Both preventive and educational acts, a general follow-up, especially to have a comprehensive care…, a clinical care too.”* After the statement of the ANP’s mission, the responders unanimously imagined them as part of a multidisciplinary team, or even within a partnership between physician and ANP. The implementation of the ANPs should be discussed. *P1 “It is in our interest to develop this activity. It’s up to us physicians to help as much as possible the integration of nurses in advanced practice into our teams.” P6 “*…*there is a complementary aspect that we need to work on, which is to add non-medical care in chronic pathologies to improve patients’ knowledge.”* All responders described the ANP’s activity as complementary to the medical activity.

#### 3.3.2. But this new status may also represent a “threat” to other professions

According to respondents, one of the challenges ANPs would face is to not infringe on existing professions. The presence of expert nurses can be confusing for some practitioners: *P1 “We already have a nurse wound team and a nurse diabetes team. In my opinion, these two teams already almost have ANP activities.” P5: “*…*the oldest of our nurses who have the practice but not the diploma could almost act as an ANP.”*

For some physicians, the ANP will encroach on the vascular physician’s job: *P8 “He will necessarily perform activities that would otherwise be done by the vascular physician.”* … *“So yes, he encroaches, but I do not necessarily put a pejorative connotation on that. Unless there was a financial issue behind it, but here, we are working in a public hospital.”*

Vascular medicine is, for some responders, a complicated discipline due to the profile of the patients (requiring multiple skills). The ANP should therefore be able to manage a large variety of cases. Trust is crucial, and one physician seemed rather hesitant to collaborate and suggested a trial period. For nurses, the feeling of encroachment in the pejorative sense seems absent, mentioning on the contrary the support that the ANP would provide. However, one of the nurses raised an interesting point about encroaching. *N8 “*…*maybe some ANP who are particularly trained in therapeutic education may find it difficult to find the limits.”* This problem of positioning is not specific to ANPs according to one of the nurses interviewed but is present for each additional skill acquired by the nurses.

There is a need for time to clarify the function and missions of the ANP: *N3 “It’s not entirely clear and I think that as we do not have the experience; it’s once again a matter of finding one’s place by clearly defining the roles.”*

### 3.4. A mission of coordination and expertise through specific skills

The potential benefits according to the participants of an ANP in vascular medicine are listed in Table 2. When we discussed the potential risks associated with PAD patients’ care by an ANP, responders mentioned the importance for the ANP to know his or her limits to avoid decisions that would go beyond his or her field of competences. Responders acknowledge that this does not specifically apply to the status of ANP: *P1 “I do not think there is any particular risk specific to the profession of ANP for patients.”*

**Table 2:** Summary of the benefits of an Advanced Nurse Pactitionner (ANP) within the ‘vascular team’.

For the patients	
More time available	• Increased time for patients with chronic conditions.
	• Allows the consultation to focus on other elements than the drug prescription.
	• Improved service delivery by allowing more time than strictly medical time.
	• Allowing more time for therapeutic education of patients.
A more personalized follow-up	• Improvement of the monitoring of chronic pathologies (maintenance of a clinical database, thus contributing to better monitoring).
	• Follow-up is easier, closer, more personalized, more regular, explained by the proximity and the preferred relationship that can exist between nurses and patients. Thus, the physicians interviewed consider that it is easier to transmit messages through an ANP.
	• Ensures that all treatments are prescribed at the maximum tolerated dosage for treatments requiring titration.
	• Rapidly identify, alert, and refer patients in the case of adverse event.
	• Allows you to focus on the people and not only on its pathology.
	• Trust established between the ANP and the patient, allows an improved motivation to take care of oneself, promotes patient adherence to treatment (dietary rules and medication compliance).
Constant active link with patients	• Optimizing the care pathway (the ANP being intermediate between the patient and the physician, allowing to make the link and to ensure coordination missions between all the professionals working around the patient).
For the medical team	
Support for physicians	• Support for physicians who are often overworked, especially when it comes to patients with chronic conditions.
Health cost-saving	• Economic benefit: reduced medical consumption due to less costly paramedical services. Some physicians consider that providing the same quality of care at a lower cost is desirable: selected patients could benefit from ANP follow-up because some medical consultations could be replaced.
	• To increase the number of patients followed up, especially in first-time consultations, and to free up time for emergencies.
	• Better efficiency for vascular physicians in private practice setting who consider that due to the financial need to increase consultations, patient follow-up is time limited. Thus, the ANP could take care of time-consuming tasks, freeing up medical time and thus increasing the number of patients.
A reduction in the workload	• Reducing workload, especially in the follow-up of patients with chronic pathologies.
	• Helping with other tasks that can be equally time-consuming, such as clinical examination.
	• Redistribution of the workload between the ANP and the physician, thus allowing for an increase in the number of patients followed.
For the nursing team	
Team support	• Support for nurses willing to be involved in research, project work, and publication.
Educational support	• Development of skills and knowledge of young graduates and newcomers through ANP teaching.
	• Maintaining the nursing skills of the service in case of a high nurse turnover.
Link between physicians and nurses	• Better involvement of nurses in patient care (results of examinations, diagnosis, treatments, outcomes, …) by reinforcing the concept of “vascular team.”

ANP, advanced nurse practitioner. All the corresponding responses are in supplementary material.

The nurses seem to be confident that ANP will have tasks and responsibilities commensurate with their skills and that they will be able to call on a physician with whom they can collaborate when the limits of their skills are reached. It should be noted that one of the physicians stated that it would be worthwhile to control the activity of ANPs through protocols, to get standardization of their mission: *P7 “*…*it’s a bit like the residents, when a resident sees a patient, there is a senior physician who has to be available if needed.”* During the interviews, one of the physicians clearly stated that he did not want ANP to make changes in the treatments of his/her patients. On the contrary, physicians aware of the ANP status consider they are trained for that and have the knowledge to work within the limits of their competence.

### 3.5. Consultation: A key role for the ANP

For all responders, the role of ANP is mainly in consultation, especially for patients with PAD. Physicians’ proposals for the place of ANP in consultation are presented in Table 3 and Supplementary Data 3. On the other hand, the role of ANP in the hospital seems less certain for those interviewed for whom hospitalization is sufficiently rich in time dedicated to patients: *N7 “I think that the patient, in the hospital, is surrounded by a lot of different professionals. As a result, I find it a bit difficult to see the role of the ANP in this place.”*

**Table 3:** Proposals of the physicians regarding the place of an Advanced Nurse Practionner (ANP) in the consultation.

Type of consultation	Quotations of the interviewees
Consultation in two sections, with a medical section dealing with acute medical problems and an ANP section dealing with more global follow-up	P4: “A scheme that could be developed.” “A kind of two-part consultation.”
Coupled consultation (physician/ ANP)	P4: “Consultations in pairs…for the patient, it is a real added value.”
Emergency consultation: for patients with a known vascular pathology requiring an urgent appointment	P2: “…also some emergency consultations, for patients who come to the emergency department with vascular disease,” “…and also being able to do post-emergency room consultations…”
Follow-up consultation with a more repeated rhythm than medical consultations, including the management of cardiovascular prevention	P6: “there can be individual consultations with the ANP,” “…the whole secondary prevention part for the patients…,” “…there are blood pressure targets, LDL-cholesterol targets, smoking cessation, regular physical activity, therefore weight loss, weight adjustment and health and diet rules…,” “…Patients with peripheral arrtery disease need to be checked very frequently. “We need to see them, re-examine them, set objectives, try to prioritize them, find out which one objective is achieved first, come back to it because in the end, nothing has happened in a month.” “…another objective is the guidance, to give more explanation about the therapeutics.”
	P7: “Wound assessment and patient follow-up, adaptation of protocols.”
	P10: “perhaps for screening; I would see the place of an ANP in the context of screening in the general population,” “…I would see the place of the ANP more in primary prevention.”
First consultation for an unknown patient	P3: “…what would make things easier for me is if the ANP had seen the patient between the general practitioner and me,” “if there was some prior assessment work that is supposed to be done by the general practitioner but that the general practitioner does not have the resource to perform.”
	P6: “the benefit would be to be less stringent, to have a first filter with adapted prescriptions for complementary examinations or a reorientation of the patient,” “with the aim of advancing in the management before the first consultation with a physician.”
	P7: “…referring physicians who would perhaps refer to advanced practice nurses who consult for cardiovascular assessment.”
Medical-surgical consultation	P6: “…before surgery, there are many changes to be carried out, such as diabetes management to reduce surgical site infections, blood pressure management, smoking cessation, and early encouragement to increase physical activity, to improve post-operative mechanical ventilation weaning.

Pn refers to physician number *n* in Table 1. ANP, advanced nurse practitioner; LDL-cholesterol, low density lipoprotein cholesterol; and PAD, peripheral artery disease.

For some of the responders, there is, however, a place for ANP in the hospital setting, both to begin monitoring patients and to provide expert advice at the request of health care teams, and in the field of training.

## 4. Discussion

**Figure 2:**
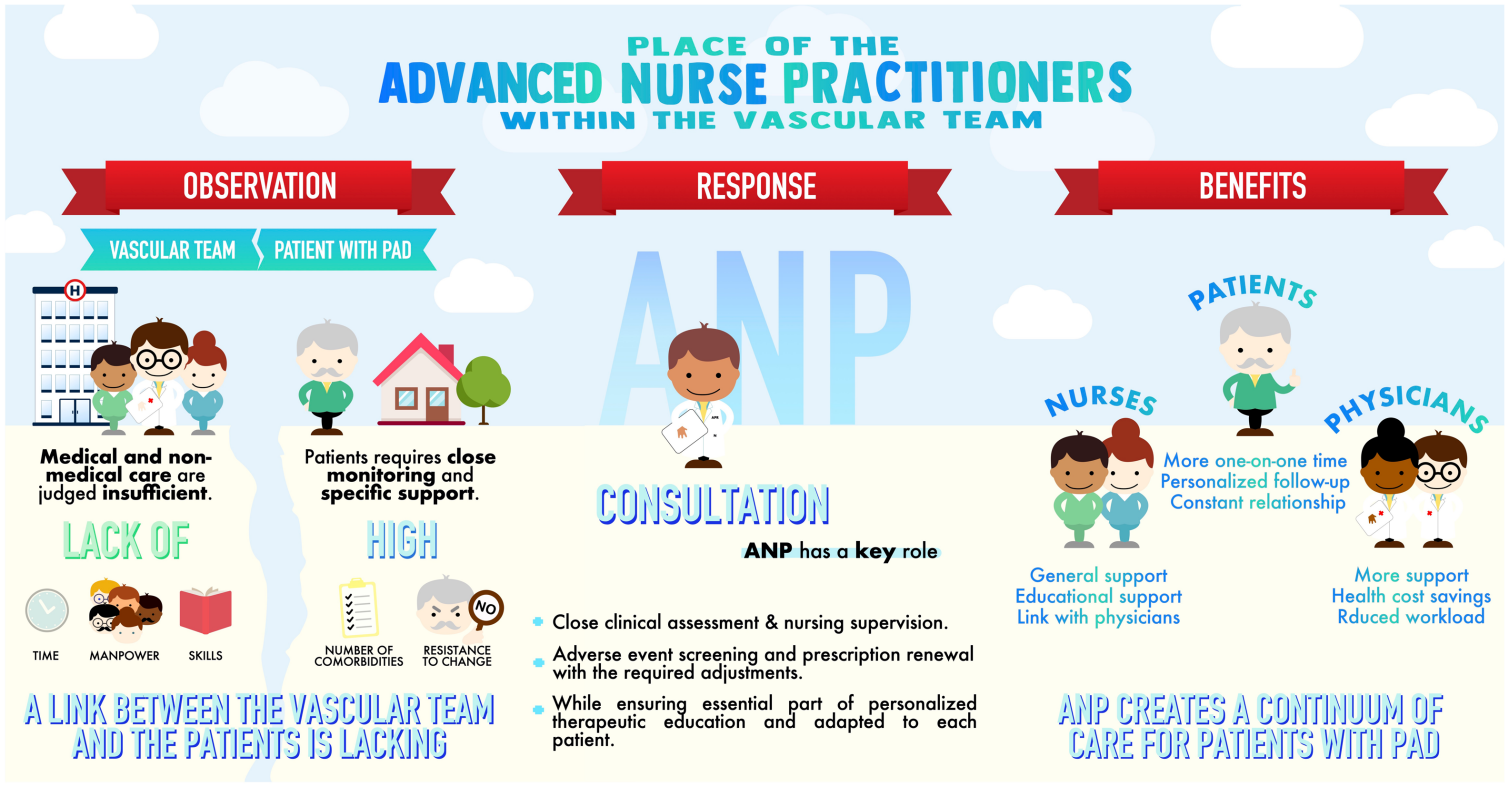
Features emerged from participants’ responses: the specific needs of vascular medicine in relation to the patients’ profiles, the place of an Advanced Nurse Practitionner (ANP) in the vascular team, and the expected benefits.

From our exploratory qualitative study dedicated to the ANP in the vascular team, three main features emerged from responders’ feedback (Figure 2):

1. Vascular medicine has a specific organization with a significant lack of time and staff to fulfill its mission because of the severity of the patients’ condition.
2. ANP is wanted to fill part of this gap. The expected benefits are a smoother care pathway and increased capacity for cardiovascular education and prevention.
3. There are some pitfalls to avoid when integrating such new practitioners within vascular teams already in place.

Vascular medicine has a limited number of practitioners in France and worldwide, in both private practice and hospital settings. In parallel, the number of patients with cardiovascular diseases is rising. Patients with PAD often have a combination of cardiac and vascular pathologies. They must meet strict secondary cardiovascular prevention targets and need close follow-up. Responders in our study have noticed several gaps in managing these patients. The time dedicated to consultations with these patients is too short, and the period between consultations is too long for an optimal follow-up. The lack of therapeutic education seems to be due to a scarcity of time, insufficient training, and according to some physicians, a lack of interest. There is also a shortfall of knowledge from some non-specialists around the goals to achieve according to recent guidelines. Lastly, cooperation between vascular medicine, vascular surgeons, and other specialists could be optimized. Four studies demonstrated that physicians only implemented recommendations 36–57% of the time, and that less than 50% of these professionals performed comprehensive risk assessments (15–18). The reasons given by physicians are the large variety of guidelines, potentially unrealistic targets, a preference for using their own experience, and a lack of knowledge about comprehensive risk assessment (19). It is particularly true for patients with PAD. Only about a third of patients with PAD in the US receive guideline-recommended medical therapy or smoking-cessation counseling, and even fewer are advised about diet and exercise (20, 21). In our study, responders also highlighted that those patients with PAD were frail and had a specific psychosocial profile that could, in part, explain the fact that they are undertreated. Concerning the psychological aspect, Striberger et al. highlighted in a qualitative study that “patients with PAD shape their own understanding of their condition” and that these beliefs can influence management and, notably patient compliance (22).

### 4.1. ANP’s role and expected benefits

According to the participants, the ANP would allow for an increase in consultation time for each patient. He or she is perceived as having a different and preferred relationship with the patients, allowing a well-fitted approach to chronic pathologies, and could be a resource for junior nurses. The intermediate position between medical and non-medical professions certainly contributes to strengthening this link. Along with coordinating nurses, they could significantly contribute to facilitating patient care pathways. This study reinforces the conclusions of a systematic literature review on nursing care in vascular surgery (23). The data shows that specialized nursing care is associated with a significant improvement in postoperative outcomes, optimization of the patient care pathway, and reduction in health care costs.

ANP’s role would, for the participants, be primarily in consultation with the patient’s follow-up as a main task. ANPs effectively stratified cardiovascular risk and aggressively managed risk factors, resulting in a significant reduction in cardiovascular complications when comparing a cohort of patients followed by ANPs with patients receiving conventional follow-up (24). Hospitalization may not be the preferred environment for ANP, at least not as much as consultation, except for Day hospitals and rehabilitation departments where there is a lack of nursing time to provide follow-up care for patients. Simmons et al. found that a nurse practitioner–led project in a vascular surgery ward resulted in improved physical activity in patients with claudication (8). Hospitalization time would be appropriate to make contact and set up a follow-up consultation. Regarding the medico-economic aspect, ANP could help minimize physician visits while improving care and reducing inappropriate consultations.

### 4.2. Barriers and challenges to successful implementation

Recognition of nursing expertise is both an advantage and a challenge for future ANPs. The lack of knowledge about ANP among healthcare teams has been described as a barrier to ANP’s inclusion (25). Physicians who have never worked with an ANP are indeed less inclined to include them and express difficulties in planning for it, particularly in the delegation of medical tasks. It is difficult to conceive that a nurse renews treatments or tapers dosages. Conversely, physicians who are aware of the role of ANPs or have already worked with them do not seem to have these barriers. They were happy to have a trained professional to carry out time-consuming tasks such as renewing therapies and modifying dosages while also providing some therapeutic education. Because of its field of competencies, ANP will have to perform acts previously dedicated to physicians and shared with nurses. ANPs will have to find their place and rely on the support of the whole vascular team members to succeed.

### 4.3. Limitation

We must consider the risk of selection bias: the physicians and nurses recruited were part of a small professional network with shared work experience. The number of practice settings limits this bias. There is also a risk of information bias since the interviewer of this research project is an ANP student. This could have led to a limitation of some negative aspects. To limit this bias, it was stated clearly at the beginning of the interview that the interviewer was not there as a future ANP. Moreover, only sincere, and uninhibited answers were sought.

Lastly, we must emphasize that this is an exploratory study whose objective was to question vascular medicine professionals in order to receive their opinion on a problem and to propose avenues that will have to be evaluated in a second phase. Thus, we have chosen to take the answers as unique responses and truths for the professionals interviewed. It seems important to us to specify that caution must be exercised when interpreting the answers of our participants.

### 4.4. Perspectives

This work opens research perspectives to carry out an observational study after the implementation of ANPs, using the elements cited by the caregivers. Namely, we retain the time dedicated to each patient, nursing projects, and the achievement of cardiovascular prevention objectives, and finally healthy lifestyle changes. We could also seek to compare patients’ satisfaction between care pathways with and without ANP and therefore assess the benefits of the implementation of ANP in a vascular team. It appears that the organization of care in different countries modifies the follow-up of patients with PAD. Indeed, vascular medicine is a medical discipline that is not represented in all countries of the world. Thus, in some countries, cardiologists care for these patients, without forgetting vascular surgeons and general practitioners. Therefore, it would be interesting to question these different professionals to know their problems and their opinions on the place of ANP in the management of patients with PAD. Certainly, the results can only be applied in health care systems where nurse practitioners and physicians specializing in managing patients with PAD (vascular surgeon, vascular physician, cardiologist, and sometimes general practitioner) work together. However, we can point out that advanced nursing practice corresponding to the criteria of the International Council of Nurses (ICN) is present in many countries, including the United States, where there are more than 325,000 nurse practitioners with a state license. Moreover, the lack of medical manpower and the need for close and personalized follow-up of patients with chronic pathologies is not specific to France.

#### What’s new

1. ANP place would be primarily in consultation and patients’ follow-up could be the main mission; and dedicated to a personalized, holistic, and modular follow-up according to the patient’s objectives.
2. ANP integration in a vascular team implies information to physicians and nurses. Only time and experience will allow creating the trust relationship essential to the formation of this new physician-ANP binomial.
3. Achievement of cardiovascular prevention goals, improvement in the patient’s quality of life, and patient satisfaction should be the expected benefits of an ANP implementation.

## Data availability statement

The raw data supporting the conclusions of this article will be made available by the authors, without undue reservation.

## Ethics statement

Ethical review and approval was not required for the study on human participants in accordance with the local legislation and institutional requirements. The patients/participants provided their verbally informed consent to participate in this study.

## Author contributions

TK, JS, and GG conceived and supervised the study. TK collected the data. TK, TM, AD, and GG wrote the manuscript. All authors contributed to the article and approved the submitted version.

## Conflict of interest

The authors declare that the research was conducted in the absence of any commercial or financial relationships that could be construed as a potential conflict of interest.

## Publisher’s note

All claims expressed in this article are solely those of the authors and do not necessarily represent those of their affiliated organizations, or those of the publisher, the editors and the reviewers. Any product that may be evaluated in this article, or claim that may be made by its manufacturer, is not guaranteed or endorsed by the publisher.
