# Supplementary material for: Role of the advanced nurse practitioner within the vascular team: A qualitative study of vascular physicians and nurses
Source: Front Public Health. 2023 Mar 30;11:1070403. doi: 10.3389/fpubh.2023.1070403 (PMC10098095; doi:10.3389/fpubh.2023.1070403)
Supplement: Supplementary file 1 [file Table_1.docx]

# **Supplementary Data 1. The semi-structured interview guide**

Interview No: ......... Date: .... /.... /2021 Start time: ....... / End time: .......

*My name is Thibaut KUBIAK, I am a nurse and student in master 2 at the University of Paris. I am currently preparing a diploma of Advanced Nurse Practitioner (ANP) mentioning chronic multiple pathologies, prevention, and common multiple pathologies in primary care. Within the framework of this master's degree, I am doing a dissertation.*

Physician / Nurse

Degrees: ......................................................................................................................................................................................................................................................................................................................................

Sex : Female / Male. Age : ......... years. Number of years of practice : ......................

**Questions :**

**PART 1: ANP**

1. Did you know about the ANP profession before this interview? If yes, what did you remember about the job (function, law, level of education, skills, have you ever worked with ANPs? If yes, in what context?

🡪 If no: *The ANP has acquired the theoretical knowledge, complex decision-making skills, and clinical skills required for advanced practice.*

*The decree specifies that advanced practice includes:*

- *Orientation, education, prevention, or screening activities*
- *Acts of evaluation and clinical conclusion, technical acts and acts of clinical and paraclinical surveillance*
- *Prescriptions for health products not subject to medical prescription, prescriptions for complementary tests, renewals, or adaptations of medical prescriptions.*

*By acquiring skills in the medical field, he/she follows patients who are entrusted to him/her by a physician, with his/her agreement and that of the patients. He/she will regularly see these patients for the follow-up of their pathologies, according to the conditions planned by the team.*

*The ANP will discuss the patients' cases during the regular exchange, coordination and consultation times organized with the team. He/she will return to the physician when the limits of his/her field of competence are reached or when he/she identifies a deterioration in a patient's health status.*

1. In your opinion, why did the legislator create this new profession in France?
2. What would be your vision of the place of the APN in the structures in which you evolve?

- Position?
- Missions?
- Place in the team?

1. On a scale of 1 to 10, how much would you rate your desire to work with ANPs? (1 = I am against working with an ANP, 10 = I am absolutely for working with an ANP) and why?

**PART 2: ANP and PAD**

*We propose to integrate the ANP into a vascular medicine team. The ANP would perform consultations in coordination with the vascular physician and the treating physician to ensure close follow-up of patients with PAD, including management of secondary prevention of cardiovascular risk factors. The ANP will participate in discussions with the team regarding the management of the patient.*

*The objectives would be, in accordance with the recommendations of the learned societies, to:*

- *Allow early detection of complications.*
- *Monitor the patient between medical consultations and nursing care.*
- *To adapt drug and non-drug management within the scope of the ANP's competence.*
- *Performing educational activities without replacing the nurses.*

1. What do you think about it?

- What would be for you the benefits that this type of care could bring?
- What would be the risks, on the contrary?

1. Concerning your job and your missions with these patients, do you consider that the ANP encroaches on your own activities?

- If yes, which activities?

1. Do you have any suggestions for this type of ANP follow-up?
2. In your practice, have you identified any difficulties, shortcomings or needs in the management of patients with PAD? Which ones?

- In the city? In the hospital?

1. In your opinion, how could the management of patients with cardiovascular risk factors be improved?

- In hospital?
- In the city?

1. Do you have any unaddressed items that you would like to address?

**Supplementary data 2. The Consolidated Criteria for Reporting Qualitative Studies (COREQ) list.**

| **Topic** | **Item No.** | **Guide Questions/Description** | **Reported on Page No.** |
| --- | --- | --- | --- |
| **Domain 1: Research team and reflexivity** | | | |
| *Personal characteristics* | | | |
| Interviewer/facilitator | 1 | Which author/s conducted the interview or focus group? | II. Methods - 3. In-depth Interviews |
| Credentials | 2 | What were the researcher’s credentials? e.g. PhD, MD | - TK: Msc - ANP (Student during the interviews)  - GG: MD, PhD  - JS: MD, Msc |
| Occupation | 3 | What was their occupation at the time of the study? | IV. Discussion - 3. Limitation |
| Gender | 4 | Was the researcher male or female? | Male |
| Experience and training | 5 | What experience or training did the researcher have? | First experience, MSN, help from people who have already conducted qualitative research |
| *Relationship with participants* | | | |
| Relationship established | 6 | Was a relationship established prior to study commencement? | II. Methods - 3. In-depth Interviews |
| Participant knowledge of the interviewer | 7 | What did the participants know about the researcher? e.g. personal goals, reasons for doing the research | II. Methods - 3. In-depth Interviews, IV. Discussion - 3. Limitation, |
| Interviewer characteristics | 8 | What characteristics were reported about the inter viewer/facilitator? e.g. Bias, assumptions, reasons and interests in the research topic | IV. Discussion - 3. Limitation |
| **Domain 2: Study design** | | | |
| *Theoretical framework* | | | |
| Methodological orientation and Theory | 9 | What methodological orientation was stated to underpin the study? e.g. grounded theory, discourse analysis, ethnography, phenomenology, content analysis | II. Methods - 4. Data Analysis |
| *Participant selection* | | | |
| Sampling | 10 | How were participants selected? e.g. purposive, convenience, consecutive, snowball | II. Methods - 2. Selection of participants and consent |
| Method of approach | 11 | How were participants approached? e.g. face-to-face, telephone, mail, email | II. Methods - 2. Selection of participants and consent |
| Sample size | 12 | How many participants were in the study? | III. Results, Table 1 |
| Non-participation | 13 | How many people refused to participate or dropped out? Reasons? | 23 persons were solicited to participate in the study. 3 physicians did not respond to emails, 1 was unavailable, and 1 physician declined to participate because he did not wish to be recorded. |
| *Setting* | | | |
| Setting of data collection | 14 | Where was the data collected? e.g. home, clinic, workplace | II. Methods - 3. In-depth Interviews |
| Presence of nonparticipants | 15 | Was anyone else present besides the participants and researchers? | II. Methods - 3. In-depth Interviews |
| Description of sample | 16 | What are the important characteristics of the sample? e.g. demographic data, date | II. Methods - 2. Selection of participants and consent |
| *Data collection* | | | |
| Interview guide | 17 | Were questions, prompts, guides provided by the authors? Was it pilot tested? | II. Methods - 3. In-depth Interviews |
| Repeat interviews | 18 | Were repeat inter views carried out? If yes, how many? | No |
| Audio/visual recording | 19 | Did the research use audio or visual recording to collect the data? | II. Methods - 3. In-depth Interviews |
| Field notes | 20 | Were field notes made during and/or after the interview or focus group? | Yes |
| Duration | 21 | What was the duration of the inter views or focus group? | III. Results, Table 1 |
| Data saturation | 22 | Was data saturation discussed? | III. Results |
| Transcripts returned | 23 | Were transcripts returned to participants for comment and/or correction? | No. This was not the methodology of the study. In fact, the post-interview modification does not allow the spontaneity of the comments collected to be maintained. |
| **Domain 3: analysis and findings** | | | |
| *Data analysis* | | | |
| Number of data coders | 24 | How many data coders coded the data? | II. Methods - 4. Data Analysis |
| Description of the coding tree | 25 | Did authors provide a description of the coding tree? | Thematic tree corresponds to the results |
| Derivation of themes | 26 | Were themes identified in advance or derived from the data? | II. Methods - 4. Data Analysis |
| Software | 27 | What software, if applicable, was used to manage the data? | II. Methods - 4. Data Analysis |
| Participant checking | 28 | Did participants provide feedback on the findings? | No |
| *Reporting* | | | |
| Quotations presented | 29 | Were participant quotations presented to illustrate the themes/findings? Was each quotation identified? e.g. participant number | Yes |
| Data and findings consistent | 30 | Was there consistency between the data presented and the findings? | Yes |
| Clarity of major themes | 31 | Were major themes clearly presented in the findings? | Yes |
| Clarity of minor themes | 32 | Is there a description of diverse cases or discussion of minor themes? | Yes |

MD: Doctor of Medicin; PhD: Philosophiæ doctor ; Msc: Master of Science ; ANP: Advanced Nurse Practitioner.

## Supplementary data 3. Quotations of physicians and nurses concerning the benefits of having an Advanced Nurse Practitioner within the ‘vascular team’ (associated with Table 3 in the manuscript).

| **For the patients** | |
| --- | --- |
| **More time available** | - P2 ‘To take the time, for example 1 hour in consultation with a patient who has several chronic, ‘... to take more time with patients, which physicians are doing less and less. ‘ - N7 ‘To be able to really concentrate on the patient, only on him, on his pathologies, for the follow-up of the disease’. - P7 ‘It would also allow consultations to be a little longer and more patient-oriented than prescribing medication.’ - P2 ‘We make ourselves a little more useful by allowing, for example, the implementation of an ANP who would see patients in consultation every 3 months rather than every 6 months.’ - P9 ‘Educate as much as possible because the physician never spends enough time.’ - P4 ‘...it allows for a really much more satisfactory management in terms of therapeutic education.’ |
| **A more personalised follow-up** | - P4 ‘To allow better follow-up of the patient outside of specialized consultations’, - P7 ‘It would allow good overall management’, - P2 ‘To take stock of everything, to really see the patient as a whole’. - P2 ‘Doing consultations with therapeutic education, even a little more personalized. - P1 ‘Follow up patients more closely in certain pathologies. - P4 ‘we could have closer follow-up’. - P7 ‘This would allow for more regular follow-up, easier access and because, in private practice, we are completely overwhelmed.’ - P8 ‘The nurse has more of a check-list mindset than the physician’. - P2 ‘When there is a deterioration in the patient's health, it allows them to be seen a little earlier. - P1 ‘It also allows us to answer questions from patients who often find it much easier to talk to nurses than to physicians. There is a kind of barrier between the patient and the physician, even for patients we have known for a very long time. ... ‘They don't answer me in the same way...’ ... ‘... when we talk about hygiene and dietary measures, for example. |
| **Constant active link with patients** | - P4 ‘It just lacks that continuum that could really optimize the treatment and management of patients.’ - P8 ‘For the patient with PAD, the doctor is not going to systematically think about doing the assessment of all the locations of atherosclerosis. It's not the ANP's job to do it, but in any case, it's the ANP's job to coordinate it, they can help coordinate it, - P1 ‘the ANP makes it possible to make the link between everyone, between the general practitioner, the specialist and the patient.’ |
| **For the medical team** | |
| **Support for physicians** | - P4 ‘We have so much work, I think it would relieve us’. - P5 ‘... Relieve the general practitioners and cardiologists of a certain number of consultations while leaving them space, on chronic pathologies which can be quite energy consuming.’ |
| **Health cost-saving** | - P8 ‘... if I were the hospital administrator, I would find it interesting to be able to have care of the same quality at a lower price’, P6 ‘The main aim of the government is to reduce medical consumption by offering paramedical services’. ... ‘Afterwards they will see if it costs too much to reduce elsewhere, but it is obviously a step forward, at least in the management of chronic pathologies.” - P8 ‘I don't know if I can say less work for us physicians. Because as there will be an increase in the number of patients...’, P6 ‘it will open us up to other patients that we can't see at present. Increase the number of first times consultations’. - P9 ‘Relieving the physician and the time spent explaining things anyway.’... to be able to space out the consultations as well, to see the patient a little less regularly, to open the door to other emergencies ‘. |
| **A reduction in the workload** | - N8 ‘... to relieve the physicians a little because of the experience and expertise of the ANP ‘. - P2 ‘... all the procedures too, I'm thinking for example of taking general practitioners. It can also help in terms of saving time, to have advanced practice nurses who know how to do it and who have the training to do it. |
| **For the nursing team** | |
| **Team support** | - N1 ‘an ANP in the vascular medicine department, that seems very interesting to me, it would be necessary to communicate on this’ ... a driving force in the department, that's it’, ‘... There are many nurses who have expertise in vascular medicine, who do a lot of things, but this is never valued’. |
| **Educational support** | - N5 ‘Develop skills, knowledge ... ‘, ‘... ANP could provide knowledge to the nursing team ... ‘. |
| **Link between physicians and nurses** | - N2: ‘to inform the team of what is going to be planned for the patient and to anticipate the rest of the care.” - P2: ‘... to get closer to certain care in neighboring countries which already have more or less this approach, where there is less of a split between nurses and physicians.’ |

ANP: Advance Nurse Practitioner; PAD: Peripheral artery disease; P: physicians; N: nurses
